# The Impact of Realistic Age Structure in Simple Models of Tuberculosis Transmission

**DOI:** 10.1371/journal.pone.0008479

**Published:** 2010-01-07

**Authors:** Ellen Brooks-Pollock, Ted Cohen, Megan Murray

**Affiliations:** 1 Department of Epidemiology, Harvard School of Public Health, Boston, Massachusetts, United States of America; 2 Division of Global Health Equity, Brigham and Women's Hospital, Boston, Massachusetts, United States of America; 3 Division of Infectious Diseases, Massachusetts General Hospital, Boston, Massachusetts, United States of America; McGill University, Canada

## Abstract

**Background:**

Mathematical models of tuberculosis (TB) transmission have been used to characterize disease dynamics, investigate the potential effects of public health interventions, and prioritize control measures. While previous work has addressed the mathematical description of TB natural history, the impact of demography on the behaviour of TB models has not been assessed.

**Methods:**

A simple model of TB transmission, with alternative assumptions about survivorship, is used to explore the effect of age structure on the prevalence of infection, disease, basic reproductive ratio and the projected impact of control interventions. We focus our analytic arguments on the differences between constant and exponentially distributed lifespans and use an individual-based model to investigate the range of behaviour arising from realistic distributions of survivorship.

**Results:**

The choice of age structure and natural (non-disease related) mortality strongly affects steady-state dynamics, parameter estimation and predictions about the effectiveness of control interventions. Since most individuals infected with TB develop an asymptomatic latent infection and never progress to active disease, we find that assuming a constant mortality rate results in a larger reproductive ratio and an overestimation of the effort required for disease control in comparison to using more realistic age-specific mortality rates.

**Conclusions:**

Demographic modelling assumptions should be considered in the interpretation of models of chronic infectious diseases such as TB. For simple models, we find that assuming constant lifetimes, rather than exponential lifetimes, produces dynamics more representative of models with realistic age structure.

## Introduction

Mathematical models of tuberculosis (TB) are important tools for investigating the dynamics of epidemics and identifying strategies for disease control [1]–[6]. Models have guided the choice of case finding strategies, treatment approaches, and identified operational targets aimed at achieving TB elimination [5], [7], [8]. The rise of the HIV epidemic [9] and the emergence of highly drug-resistant *Mycobacterium tuberculosis* [7], [10] complicate TB control and emphasize the need for accurate mathematical descriptions that can be used to project the potential effects of new diagnostics, therapeutic approaches or vaccines [11]–[13].

Transmission of TB occurs when people with infectious pulmonary disease cough, sneeze, talk or otherwise aerosolize tubercle bacilli which may be inhaled by others. Among those infected, approximately 5% develop active disease within the first few years but the majority remain asymptomatically infected. Of these, 5–10% are expected to reactivate their latent infection and develop TB disease later in life [14], [15].

Most mathematical models of TB transmission classify individuals as either susceptible (S), exposed (E), infectious, (I) or recovered (R) [16]. Previous work has focused on capturing the complex natural history of TB and consequently, most current models include a variation on a standard set of compartments [2]–[4], [17]. In contrast, there has been little attention and no consensus on the inclusion of demographic patterns that determine age structure. Since the time course of TB infection is of the same order as the human lifespan and the majority of infected individuals eventually die of causes unrelated to TB, demographic effects are likely to be important for predictions about control interventions.

Compartmental *SEIR* models capture demographic trends by assigning rates of births and age-specific mortality. Despite being unrealistic for most human populations [18], a constant mortality rate is a widely accepted simplification because it has a minimal impact on the dynamics of acute infections [16], [19], which is largely because the infectious period is not affected by assumptions about non-disease related mortality [20], [21]. Considering realistic demography has been shown to alter the average age of infection [22], [23], although the explicit effect of age structure on chronic disease transmission has not been investigated.

In this paper, we explore the impact of age structure on TB model dynamics. We investigate the role of survivorship by comparing model outcomes using constant, exponentially distributed and Gompertz-like lifespans. Of these approaches, the Gompertz function produces the most realistic patterns of human mortality by allowing the risk of death to increase exponentially with age [24], [25]. Using a simple model that captures the essential features of TB natural history, we consider analytic approximations for the basic reproductive ratio (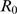) and the steady-state prevalence of infection and disease. We discuss the implications of using simplified survivorship functions by comparing the results with realistic distributions and investigate the impact of survivorship function on parameter estimation and assessments of the relative success of control interventions when models are calibrated to the same data.

## Methods

We begin by describing a simple age-structured model of TB transmission with alternative assumptions about survivorship. We use analytic approximations of the prevalence of infection and disease and the basic reproductive ratio as the basis for investigating the effect of changing survivorship in models with otherwise identical natural history assumptions. Finally, we use an individual-based model to examine how age structure may affect the projected impact control interventions. Full details of the models and equations are contained in Technical Appendix S1.

### TB Natural History

We capture the natural history of TB by classifying individuals based on age and infection status using an *SEIR* compartmental model [2], [16], [25], [26]. We assume that all individuals start life susceptible to infection without prior exposure (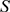) and die due to non-TB causes at an age dependent rate 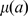. Susceptible individuals become infected at a time varying rate (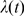) which reflects the total prevalence of disease and the probability of transmission given an encounter between an infectious case and a susceptible individual (transmission rate 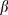). We assume that a small fraction of infected persons develops active disease (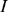) immediately after infection while the remainder develop a chronic latent asymptomatic infection (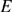). For consistency with previous TB models [2], [6], [8], [13], [17], [27], we assume that the risk of progressing from latency to active infectious disease is constant for adult ages (progression rate 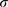). Latently infected individuals are able to be reinfected with a decreased probability representing partial immunity. Infectious individuals recover (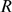) or die due to disease.

### Survivorship

**Figure 1:**
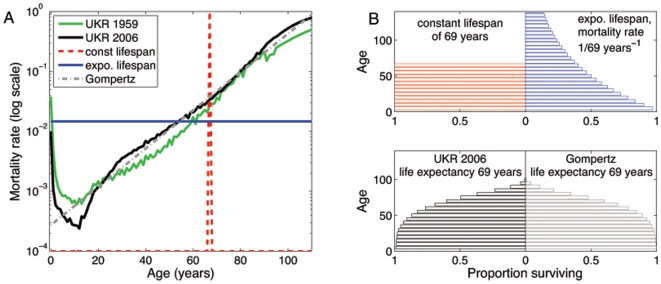
Mortality and survival functions fitted Ukraine 1959 and 2006 data. (A) The age-specific mortality rate in Ukraine in 1959 (green solid line) and 2006 (black solid line), arising from constant (dashed red line), exponential (solid blue line) and Gompertz-like (dot-dashed grey line) lifespans, fitted to Ukraine 2006 data. (B) The probability of surviving until a given age for the same three types of survival functions: constant, exponential and Gompertz-like lifespans. The probability of survival for Ukraine in 2006 is taken from the life tables in [28]. The life expectancy was 69 years and the fitted Gompertz parameters were 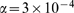 and 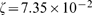.

Mortality in infectious disease models is commonly included as a constant age-independent mortality rate, 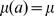 [2], [19], [30], which results in exponentially distributed life expectancies with an average life span of 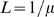 years. An alternative model is one where an individual survives until a particular age 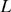 and then dies [18], [30] (Fig. 1). The survivorship function, 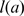, and the mortality rate, 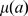, can be described in terms of age 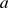 and life expectancy, 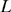:
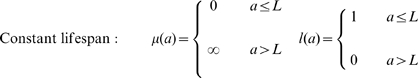
(1)

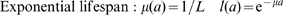
(2)where 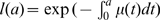 gives the probability of surviving until age 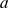. A constant lifespan underestimates early mortality, although produces an excellent approximation for populations with high infant survival [25], while an exponential lifespan overestimates mortality in younger ages and underestimates mortality in older age groups, resulting in an underestimation of early survival (Fig. 1).

Human survivorship is better characterized by a mortality rate that increases exponentially with age (Fig. 1) [18]. This type of mortality is described by a Gompertz function for positive parameters 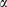 and 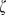:
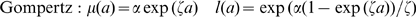
(3)


We use mortality data from Ukraine in 2006 as a country with detailed population data [28] and a substantial TB prevalence [29]. Using the mortality data, we calculated a life expectancy of 69 years and Gompertz parameters 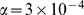 and 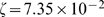 (Fig. 1B). While the Gompertz function is more realistic, we note that it does not capture infant mortality nor produce the classic bell-shaped age distribution that arises due to growing populations. Further discussion of Gompertz parameters can be found in Technical Appendix S1.

## Results

### Prevalence of Infection and Disease

TB models are usually calibrated to an observed prevalence of disease by varying the transmission parameters [2], [6], [8]. The basic reproductive ratio is generally used as a target for disease control, rather than for describing early epidemic growth rates. To quantify the effect of survivorship on inferences from epidemic models, we compared the prevalence of infection, disease and basic reproductive ratio under models with alternative descriptions of survival.

Natural mortality affects steady state dynamics in two ways: by reducing the length of time an individual is infectious and by decreasing the probability of a secondary infection progressing to active disease during their lifetime. Assuming constant lifetimes increases the lifespan for the majority of individuals and because the population size is fixed, there is slower population turnover. This affects the number of individuals in each infection state: the numbers of susceptible persons and infectious cases are increased, but the number of latently infected persons is reduced.

For given TB parameters, the proportion of the population not exposed to disease is up to 50% greater with constant lifetimes. New cases of active disease are modelled as resulting from a recent primary infection (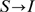) or an existing latent infection (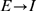). With constant lifetimes, the number of recent infections is greater due to the larger pool of susceptible individuals and the number of cases due to reactivation or reinfection is also greater due to the increased probability of progressing from latency to disease. The remainder of the population is classified as latently infected, therefore constant lifetimes result in smaller estimates of the prevalence of infection.

Rather than fixing parameters, TB models are generally fitted to an observed prevalence. We show that the type of survivorship directly affects estimates of epidemiological parameters by absorbing the difference in life-years. For a fixed prevalence, constant lifespans are consistent with lower parameter estimates than exponential lifespans. For example, if we require equal numbers of latently infected individuals to progress to disease in both models, then we must assume a smaller progression rate for the constant lifetime model. This can be seen analytically by rearranging the equations for prevalence of disease so that 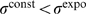 (see Technical Appendix S1). Therefore, if the two models are calibrated to the same data, individuals will progress from latency to active disease at a slower rate in the model with constant lifespans and any interventions aimed at reducing reactivation or reinfection would have a smaller impact.

### The Basic Reproductive Ratio

We use the basic reproductive ratio (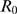) to investigate how realistic age structure affects the ease with which a theoretical TB-like disease could be eradicated in a population. The basic reproductive ratio is defined as the average number of secondary cases produced by a single infectious case and therefore encompasses the average number of secondary infections per case and the probability that a secondary infection will lead to disease. Natural mortality affects 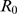 by modifying the infectious period and the probability of a secondary infection progressing to disease. 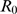 for both constant and exponential models can be calculated using the survivorship function – see Technical Appendix S1 and [33], [34].

**Figure 2:**
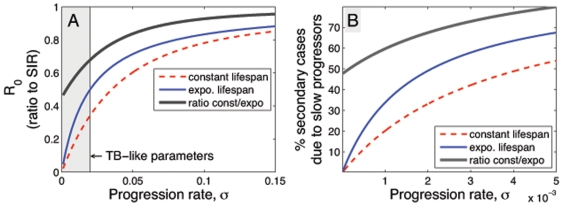
A comparison of two models with alternative assumptions about mortality. Red dashed lines: all individuals live for a constant lifetime; blue solid lines: individuals experience a constant mortality rate and have exponentially distributed lifetimes. (A) The basic reproductive ratio (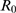) for TB as a function of the progression rate from latency to disease, 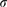. As the latent period decreases, 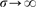, the *SEIR* models approach an *SIR* description. Conversely, as 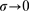, estimates from models with constant and exponential lifetimes diverge. (B) The percentage of 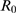 that is contributed by slow progressors to TB disease as a function of progression rate, 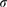. In all scenarios, the exponential model predicts that a greater proportion of disease is due to reactivation.

A model with constant lifespans results in a smaller effective infectious period, although for a mean infectious period of less than 15 years the estimates differ by less than 5%. A larger difference arises when we consider the probability of progressing from latency to active disease in each of the models. In a model with exponential lifespans, the probability of progressing from latency to active disease is up to twice as likely (Fig. 2A). This means that when we calculate 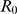 for both systems, the exponential model yields a greater value. The difference between these estimates of 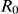 can be approximated by considering the latent period less than the life expectancy, 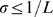, an infectious period less than the average latent period and life expectancy, 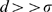 and 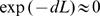 so that:
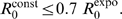



Our analysis indicates that the main effects of including realistic age structure are to alter our estimates of the proportion of people with latent infections and the proportion that are expected to progress from latency to active disease. A further manifestation is that a model with exponential lifespans will predict at least 25% more disease due to reactivation and reinfection than a model with constant lifespans (Fig. 2B).

### The Influence of Survivorship Function on the Effect of Interventions

To illustrate our results further, we fit three individual-based models, differing only in survivorship function, to the same observed data. In each case we explore the effect of control interventions by examining the level of interventions required for disease eradication. The age structure and natural history parameters for each model are determined by the type of survivorship.

**Figure 3:**
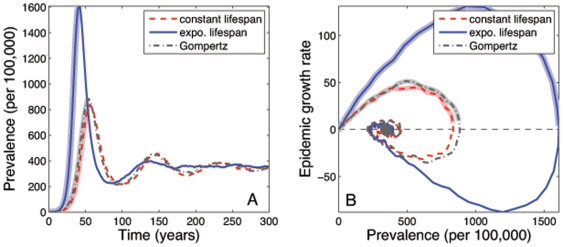
TB epidemics produced using three models with alternative assumptions about mortality. Red dashed lines: all individuals live for a constant lifetime; blue solid lines: individuals experience a constant mortality rate and have exponentially distributed lifespans; grey dot-dashed lines: mortality increases exponentially with age (Gompertz mortality). Each model is fitted to an equilibrium prevalence rate of 350/100,000 by allowing the transmission rate to vary. (Baseline parameters given in Table S1 of Technical Appendix S1). (A) The epidemic profile in time. (B) The epidemic growth rate as a function of prevalence. The initial period of epidemic growth is highlighted using the thick shaded lines in both figure (A) and (B).

We fit each model separately to Ukraine prevalence of 350/100,000 [29] by varying the transmission rate, 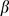 (Fig. 3A). Consistent with our analytic results, a constant lifespan yields a lower estimate of the transmission rate. On a scale between the constant and exponential estimates, the Gompertz estimate lies within 10% of the constant value:
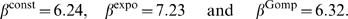



The basic reproductive ratio, calculated using equations derived in Technical Appendix S1, is greater for an exponential lifespan:
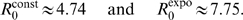



The higher value of 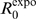 is confirmed by examining the early growth rates of the epidemics (Fig. 3B). We find that the growth rate under Gompertz mortality is better approximated by a model with a constant lifespan. In this scenario, 26% and 18% of active TB cases are due to primary progression with constant and exponential lifespans respectively. Reactivation causes 0.9% and 1.3% of cases respectively, and with these parameters, the majority, 74% and 81%, of infection is due to fast progression from latency following reinfection. These values are sensitive to the prevalence of disease and parameter estimates.

**Figure 4:**
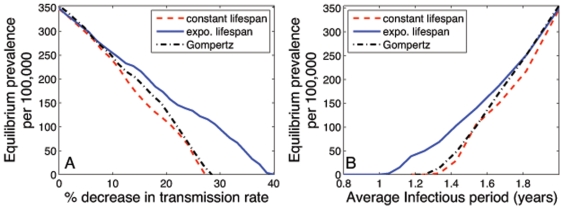
Investigation into epidemic control for a model with three types of survival. Red dashed lines: constant lifespan; solid blue lines: exponential lifespan and black dot-dashed lines Gompertz survival. In each case, we determined the necessary decrease in parameters for the prevalence to fall to zero. (A) Reduction in transmission rate, 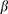, and (B) reduction in average infectious period, 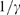. The lines denote the average of 10 model runs.

Using the above parameters, we consider the necessary reduction in transmission to eradicate disease in each of the models. All three models predict similar reductions in prevalence for decreases in transmission rate of less than 10% (Fig. 4A). The model with constant lifespans requires a 20% reduction in transmission, whereas the model with exponential lifespans requires a 30% reduction. The model with Gompertz mortality requires a 22% reduction in transmission, differing from the constant model by 15% and from the exponential model by 28%. The behaviour of the model with Gompertz survival was also quantitatively similar to the model with constant lifespans.

For reductions in the average infectious period, the model with constant lifespans requires the smallest reduction in order to achieve disease eradication. An average infectious period of 16.2 months is needed for eradication in the constant lifespan model, whereas the exponential lifespan model requires a further reduction to 12.2 months. The estimate of 15.4 months from the Gompertz model is 25 days less than the constant lifespan model estimate and 100 days greater than the exponential model estimate (Fig. 4B). Absolute reductions of the parameters have the greatest effect on prevalence in the model with constant lifespans. This has the practical effect that intervention strategies assessed through a mathematical model that assumed an exponential lifespan will predict less dramatic benefits than we actually observe when survivorship is better described by a constant lifespan.

## Discussion

Our analysis illustrates that natural (non-disease related) mortality and age structure play an important role in the dynamics of chronic infectious diseases such as TB. We explored three common characterisations of survivorship: constant or fixed lifetimes, exponentially-distributed lifetimes and lifetimes resulting from a Gompertz mortality function (mortality that increases exponentially with age). Our results indicate that the choice of survivorship strongly affects steady-state dynamics, parameter estimation and predictions about the effectiveness of control interventions.

Our findings are consistent with previous investigations into the effects of realistic infectious periods of acute diseases [20], [21]. For example, we find that constant lifetimes result in a greater prevalence of disease than exponential lifetimes. However, this difference in prevalence is exaggerated for chronic infectious diseases. For diseases with a prolonged period of latency, we also find that the number of people with latent infection and the proportion of individuals that progress from latency to active disease differ between models that assume different patterns of survivorship. In an exponential lifetime model, fewer individuals progress from latency to disease due to the variability in latent periods.

In addition to exploring the effect of mortality on steady-state dynamics, we also investigated the public health implications of mortality assumptions in mathematical models that are used to inform recommendations for disease control. We find that models that assume exponential lifetimes result in larger parameter estimates and overestimate the effect of treating TB disease and underestimate the effect of treating latent TB infection when compared to the same interventions in models with more realistic lifetimes. Quantifying the importance of particular routes to disease is important for balancing individual- and population-level control interventions that will lead to a reduction in disease burden.

Age-structure and natural mortality have not been a focus of TB models. Assumptions about survivorship are often hidden; our results suggest that these modelling assumptions should be more explicitly stated given their impact on model dynamics and results. Two types of mortality patterns are commonly used in models of TB epidemics. The majority of non-age structured TB models implicitly assume that lifetimes are exponentially distributed [2], [6], [13], [35]. More detailed numerical approaches often use vital statistics to obtain age-specific fertility and mortality rates [4], [8], [32], [36], [37]. Our work suggests that these two approaches can produce different results and that parameter estimates are not easily transferred between models with different survivorship functions. For simple models, we find that assuming constant lifetimes, rather than exponential lifetimes, produces dynamics more representative of models with realistic age structure.

The aim of our analysis was to quantify the differences resulting from commonly used modelling assumptions about natural mortality. Additional complexity resulting from altered living standards, treatment rates and co-morbidity with other diseases are important features that could potentially confound the effects of altered survivorship. We focused on TB natural history in otherwise healthy populations; the interaction between the HIV and TB affects both TB natural history and the underlying demography [38]. Our analysis would suggest that increased TB progression rates associated with HIV-TB co-infection would reduce the effects of incorrectly modelling survivorship, however including the reduction of life expectancy associated the HIV epidemic could potentially re-introduce the differences. Host demography plays an important role in the manifestation of chronic infectious diseases such as TB; incorporating the effects of survivorship into transmission models will help to understand the mechanisms of TB epidemics in ageing or growing populations.

## Supporting Information

Technical Appendix S1 Technical Appendix S1 contains details of the model equations and baseline TB parameters, further discussion of Gompertz parameters, analytic derivations of main results and discussion of model generality.
(0.07 MB PDF)
