# Supplementary material for: The Impact of Realistic Age Structure in Simple Models of Tuberculosis Transmission
Source: PLoS One. 2010 Jan 7;5(1):e8479. doi: 10.1371/journal.pone.0008479 (PMC2797602; doi:10.1371/journal.pone.0008479)
Supplement: Technical Appendix S1 — contains details of the model equations and baseline TB parameters, further discussion of Gompertz parameters, analytic derivations of main results and discussion of model generality. (0.07 MB PDF) [file pone.0008479.s001.pdf]

Technical Appendix S1: The impact of realistic age structure in simple models of tuberculosis transmission

Ellen Brooks-Pollock*, Ted Cohen, Megan Murray
*E-mail: ebrooks@hsph.harvard.edu

We describe a simple age-structured model of TB transmission with alternative assumptions about survivorship. We use analytic approximations of the prevalence of infection and disease and the basic reproductive ratio as the basis for investigating the effect of changing survivorship in models with otherwise identical natural history assumptions.

A basic model for TB transmission

TB dynamics can be captured by classifying individuals based on age and infection status [1–3]. Susceptibles become infected at a time varying rate which reflects the prevalence of infectious cases of all ages a at a given time and the probability of transmission, β, given an encounter between an infectious case and a susceptible individual,

$$\lambda = \beta \bar{I} := \beta \int_0^\infty I(a)da.$$

The equations for the proportion of the population in each infection state are:

$$\frac{\partial S(a,t)}{\partial t} + \frac{\partial S(a,t)}{\partial a} = -(\lambda + \mu(a))S(a,t) \tag{1}$$

$$\frac{\partial E(a,t)}{\partial t} + \frac{\partial E(a,t)}{\partial a} = (1-f)\lambda S(a,t) - (\sigma + \varepsilon\lambda + \mu(a))E(a,t) \tag{2}$$

$$\frac{\partial I(a,t)}{\partial t} + \frac{\partial I(a,t)}{\partial a} = f\lambda S(a,t) + (\sigma + \varepsilon\lambda)E(a,t) - (d + \mu_{TB} + \mu(a))I(a,t) \tag{3}$$

$$\frac{\partial R(a,t)}{\partial t} + \frac{\partial R(a,t)}{\partial a} = dI(a,t) - \mu(a)R(a,t) \tag{4}$$

We take the total population size as $\bar{N} = \bar{S} + \bar{E} + \bar{I} + \bar{R} = 1$. In this system, the number of births and deaths are balanced and the number of births is equal to the number of people aged zero, $N(0) = S(0,t)$,

$$S(0,t) = \int_0^\infty \mu(a)N(a,t)da. \tag{5}$$

The other boundary conditions are $E(0,t) = I(0,t) = R(0,t) = 0$. For the static population structures used in this paper, equations (1)-(4) reduce to a system of ordinary differential equations (ODEs) [2]. We investigate the behaviour of the model for parameter ranges taken from the literature (table S1, [4, 5]).

Table S1: Baseline model parameters. Parameters used in the basic TB model (equations (1)–(4)), taken from the literature [4, 5]

parameter	value
transmission rate, β	*fitted to data*
proportion of fast progressors, f	0.1
reactivation rate, σ	1.13×10^{-4}
immunity, ε	0.65
recovery rate, γ	0.2
TB mortality rate, μ_{TB}	0.3

Models of survivorship

The survivorship function ($l(a)$) and mortality rate ($\mu(a)$) for the three types of survival used in this paper can be described in terms of age a and life expectancy, L:

$$\text{Constant lifespan}: \quad \mu(a) = \begin{cases} 0 & a \leq L \\ \infty & a > L \end{cases} \qquad l(a) = \begin{cases} 1 & a \leq L \\ 0 & a > L \end{cases} \tag{6}$$

$$\text{Exponential lifespan}: \quad \mu(a) = 1/L \qquad l(a) = \mathrm{e}^{-\mu a} \tag{7}$$

$$\text{Gompertz}: \quad \mu(a) = \alpha \exp(\zeta a) \qquad l(a) = \exp(\alpha(1 - \exp(\zeta a))/\zeta) \tag{8}$$

where $l(a) = \exp(-\int_0^a \mu(t)dt)$ gives the probability of surviving until age a. A constant lifespan underestimates early mortality, although produces a reasonable approximation of a realistic survivorship curve, while an exponential lifespan overestimates mortality in younger ages and underestimates mortality in older age groups, resulting in an underestimation of early survival (Fig. **??**). We note that in populations with high infant mortality, fitting a constant mortality rate to mortality data yields a mortality rate that is greater than the reciprocal of the life expectancy. For our example of the Ukraine in 2006, infant mortality is relatively low therefore the optimal fitted mortality rate is 1/66.5 years^{-1}, which is similar to the reciprocal of the life expectancy of 1/69 years^{-1}.

The Gompertz function contains positive parameters α and ζ, which we calculated as $\alpha = 3 \times 10^{-4}$ and $\zeta = 7.35 \times 10^{-2}$ using Ukraine mortality data [6]. The intercept parameter α affects mortality for all age groups; the slope parameter ζ differentially affects mortality for older ages. The Gompertz function can be fit to mortality data by linear regression after taking the logarithm, $\log(\mu(a)) = \log(\alpha) + \zeta a$. There are a number of approaches for improving the fit of the Gompertz mortality function such as the Gompertz-Makeham equation which includes an additional constant mortality rate for all ages groups, $\mu(a) = \mathrm{c} + \alpha \mathrm{e}^{\zeta a}$ [7].

Prevalence of disease

The prevalence of disease is often used as a measure of a TB epidemic, with models calibrated so that the equilibrium prevalence is consistent observed TB notification rates [4, 8, 9]. The number of susceptibles at equilibrium for each type of survival by integrating equation (1) twice, once with respect to time to obtain the proportion of individuals aged a that are susceptible, $S(a)$, and then with respect to age to find the total proportion of susceptibles, $\bar{S}$ [10]:

$$\bar{S}^{\text{const}} = \frac{(1 - \mathrm{e}^{-\lambda L})}{\lambda L} \qquad \text{and} \qquad \bar{S}^{\text{expo}} = \frac{1}{1 + \lambda L}. \tag{9}$$

These formulae indicate that $\bar{S}^{\text{const}} > \bar{S}^{\text{expo}}$, although when $\exp(-\lambda L)$ is small the difference is negligible. However for TB, λL can be less than one, therefore the proportion of susceptibles estimated using constant lifespans can be 50% larger than the proportion estimated using exponential lifespans.

To examine contribution of reinfection and reactivation to disease, we consider active cases produced via the latent state. Very approximately, we assume the recovery rate from active TB is on the order of years^{-1}, the force of infection is on the order of decades^{-1} and the average latent period, including individuals that do not progress from latency, occurs over centuries. If we let $d \sim \varepsilon$, then $\lambda \sim \varepsilon^2$ and $\sigma \sim \varepsilon^3$. The equations for the number of infectious people produced via latency, estimated by constant and exponential lifespans, simplify to

$$\bar{I}^{\text{const}} = \frac{1-f}{dL}(1 - \exp(-\sigma\prime L)) + o(\varepsilon) \qquad \text{and} \qquad \bar{I}^{\text{expo}} = \frac{1-f}{1+dL}\left(\frac{\sigma\prime L}{1+\sigma\prime L}\right) + o(\varepsilon). \tag{10}$$

$$\text{where} \quad \sigma\prime = \sigma + \varepsilon\lambda$$

Accordingly, due to both the higher proportion of susceptibles, and the difference in lifespans, $\bar{I}^{\text{const}} > \bar{I}^{\text{expo}}$.

The remaining individuals in the population have been infected but do not currently have active disease, i.e. individuals with a positive skin test, in the exposed (E) and recovered (R) stages of disease. A model with constant lifespans will predict a smaller proportion of the population with latent infection than a model with exponential lifespans, i.e. $\bar{E}^{\text{const}} + \bar{R}^{\text{const}} < \bar{E}^{\text{expo}} + \bar{R}^{\text{expo}}$. In the exponential lifetime model, the proportion of individuals that progresses from latency to disease, either due to reactivation or reinfection, is $\alpha = \sigma\prime/(\sigma\prime + \mu)$. In contrast, the proportion of individuals that progress from latency to disease in a model with constant lifetimes is $1 - \exp(-\alpha/(1-\alpha))$. The proportion of individuals that progress in each model is equal for the extreme values where either noone or everyone progresses to disease. For all intermediate values, fewer individuals will progress from latency to disease in an exponential lifetimes model. Therefore if we used the same parameters in each model, any interventions aimed at reducing reactivation or reinfection would have a greater impact in the constant lifetimes model.

The basic reproductive ratio

The basic reproductive ratio is the average number of secondary cases produced by a single infectious case and can be calculated using the survival function, $l(a)$ [11, 12],

$$R_0 = \frac{\beta}{d} \int_0^\infty (1 - \exp(-da)) l(a) da \left(f + (1-f) \int_0^\infty (1 - \exp(-\sigma a)) l(a) da \right). \tag{11}$$

Equation (11) is the product of the transmission rate, β, the effective infectious period, $\int (1 - \exp(-da)) l(a) da$, and the probability of progressing to active disease, $\int (1 - \exp(-\sigma a)) l(a) da$. Thus, background mortality affects R_0 in two ways, by reducing the length of time an individual is infectious, and by increasing the probability of a secondary infective dying while latently infected. Applying the survivorship functions above, yields

$$R_0^{\text{const}} = \frac{\beta}{d} \left(1 - \frac{1 - e^{-dL}}{dL} \right) \left(1 - (1-f) \frac{1 - e^{-\sigma L}}{\sigma L} \right) \tag{12}$$

$$R_0^{\text{expo}} = \frac{\beta L}{1 + dL} \left(1 - (1-f) \frac{1}{1 + \sigma L} \right) \tag{13}$$

The equation for R_0^{expo} can be recognised as the standard form of R_0 [1, 13]. The difference between these estimates of R_0 can be approximated by considering the latent period less than the life expectancy, $\sigma \leq 1/L$, an infectious period less than the average latent period and life expectancy, $d >> \sigma$ and $\exp(-dL) \approx 0$ and comparing equations (12) and (13):

$$R_0^{\text{const}} \leq 0.7\ R_0^{\text{expo}}.$$

Model generality

Our analysis uses the simple scenario of a chronic infectious disease at equilibrium to illustrate the potential effects of ignoring the importance of the birth-death process. The effects that we report are due to slow disease natural history (so that the majority of secondary infections never progress to disease) and a low prevalence of disease (small λ affecting the average age of infection). The model and parameter specification are such that these results will be potentially relevant for diseases where the average age of infection is in adulthood, such as TB in other mammals, rabies [14] or dengue [15] in low prevalence settings.

Further difficulties in capturing realistic TB dynamics are the variety of risk factors that have been found to be important for infection and disease. Notably, our model does not include age-related risks of disease and for increased realism in ageing populations, we would have to include complexities such as declining incidence over time by a mechanism that reduces the number of contacts with transmission potential. Relaxing the assumption of a constant population size is likely to reveal more interesting features of TB in diverse demographic landscapes.

References

1. Blower SM, McLean AR, Porco TC, Small PM, Hopewell PC, et al. (1995) The intrinsic transmission dynamics of tuberculosis epidemics. Nature Medicine 1: 815–21.

2. Anderson R, May R (1991) Infectious Diseases of Humans. Oxford Science Publications.

3. Vynnycky E, Fine PE (1998) The long-term dynamics of tuberculosis and other diseases with long serial intervals: implications of and for changing reproduction numbers. Epidemiology and Infection 121: 309–24.

4. Cohen T, Murray M (2004) Modeling epidemics of multidrug-resistant m. tuberculosis of heterogeneous fitness. Nature Medicine 10: 1117–1121.

5. Dye C, Williams B (2000) Criteria for the control of drug-resistant tuberculosis. Proceedings of the National Academy of Sciences 97: 8180–8185.

6. Human Mortality Database (accessed 15/07/2009). University of California, Berkeley (USA), and Max Planck Institute for Demographic Research (Germany). URL `www.mortality.org`.

7. Promislow DEL, Tatar M, Pletcher S, Carey JR (1999) Below-threshold mortality: implications for studies in evolution, ecology and demography. Journal of Evolutionary Biology 12: 314-328.

8. Dye C, Williams BG (2008) Eliminating human tuberculosis in the twenty-first century. Journal of the Royal Society, Interface 5: 653–62.

9. Vynnycky E, Fine PE (1997) The natural history of tuberculosis: the implications of age-dependent risks of disease and the role of reinfection. Epidemiology and Infection 119: 183–201.

10. Dietz K (1974) Transmission and control of arbovirus diseases. Proceedings of society for industrial and applied mathematics, Philadelphia : 104—121.

11. Lloyd A (2001) Destabilization of epidemic models with the inclusion of realistic distributions of infectious periods. Proceedings of the Royal Society B 268: 985–993.

12. Heffernan J, Smith RJ, Wahl LM (2005) Perspectives on the basic reproductive ratio. Journal of The Royal Society Interface 2: 281–293.

13. Dye C, Garnett GP, Sleeman K, Williams BG (1998) Prospects for worldwide tuberculosis control under the WHO DOTS strategy. Lancet 352: 1886–91.

14. Kitala PM, McDermott JJ, Coleman PG, Dye C (2002) Comparison of vaccination strategies for the control of dog rabies in machakos district, kenya. Epidemiology and Infection 129: 215-222.

15. Cummings D, et al (2009) The impact of the demographic transition on dengue in Thailand: Insights from a statistical analysis and mathematical modeling. PLoS Medicine 6: e1000139.
